# Pain Relieving Effect of Intraoperative Chemical Splanchnicectomy of Celiac Ganglions in Patients with Resectable Pancreatic or Gastric Masses: A Randomized Clinical Trial

**DOI:** 10.1155/2020/2675940

**Published:** 2020-03-24

**Authors:** Jalal Vahedian, Amir Saraee, Massoud Baghai Wadji, Saeed Safari, Abdolhamid Chavoshi Khamneh

**Affiliations:** ^1^Iran University of Medical Sciences, Firoozgar Hospital, Tehran, Iran; ^2^Tehran University of Medical Sciences, Shariati Hospital, Tehran, Iran

## Abstract

**Background:**

Trials of intraoperative chemical splanchnicectomy during resection of pancreatic and gastric masses resulted in significant difference in a patient's postoperative pain. This study aims to determine if splanchnicectomy by alcohol neurolysis can relieve postoperative pain after gastrectomy and Whipple surgery. The study explores differences in outcomes at first four months after surgery.

**Methods:**

Fifty-eight patients with gastric and 60 patients with pancreatic resectable masses were included (28 were lost to follow-up). Each randomized in control and intervention subgroups. Intervention subgroups underwent chemical blockage of celiac ganglions by ethanol injection at both sides of suprapancreatic aorta. Participants were asked to report their pain intensity according to the Visual Analogue Scale (VAS) at specific times.

**Result:**

The overall postoperative pain of injected Whipple and gastrectomy subgroups was lower than the noninjected Whipple and gastrectomy subgroups (*p* < 0.001). The pain-modifying effect of the injection was not different between Whipple and gastrectomy groups (*p*=0.125)

**Conclusion:**

Splanchnicectomy is recommended for pain reduction after abdominal operations. *Perspective*. This article presents positive effect of intraoperative chemical splanchnicectomy during resection of pancreatic and gastric masses on postoperative pain. This is an easy, effective, safe, and inexpensive procedure recommended for all operable gastric or pancreatic masses to palliate the pain degree.

## 1. Introduction

Gastric and pancreatic cancers and their increasing prevalence are included in today's concerns of surgeons and palliative care workforce.

Gastric and pancreatic cancers are two of the most common malignancies that are mostly diagnosed at an advanced stage notably in Eastern/Southeastern Asia. Five-year survival is expected in 25% and 22% of gastric and pancreatic masses, respectively, and can be only achieved by early onset diagnosis and providing critical therapies [1–3]. Pancreatic cancer is the fourth cause of death from cancer [4].

The majority of patients in both groups will have pain requiring increasing doses of narcotic analgesics during the course of their illness.

Cases of pancreatic cancers suffer from extreme loss of appetite and considerable weight loss; they mostly represent an excruciating pain as the most disturbing and incapacitating symptom [5, 6].

The pain caused by the upper abdominal cancers may originate from visceral, somatic, or neuropathic. Sympathetic fibers responsible for visceral pain signals are located close to celiac trunk at level T12-L2, anterolateral to the aorta. Three pairs of celiac plexus ganglions are found less than 1 cm inferior to the origin of the celiac artery. Somatic and neuropathic pains may characterize involvement of adjacent organs including peritoneum or retroperitoneum [7].

Several methods of splanchnicectomy or interruption of the main pancreatic sympathetic pathway has been applied to make this pain more tolerable [8, 9].

At recent clinical trials, unilateral and bilateral splanchnicectomy were performed throughout open abdominal surgeries and thoracoscopy. Unpredictable complications and different efficacies have been reported; however, immediate pain relief and long-term improvement achieved in 80 and 50 percent of patients, respectively [10]. Some other studies achieved complete visceral pain reliefs till death in 60–75 percent of patients [9].

Celiac plexus block (CPB) was first performed in 1916. Later trials on patients with unresectable pancreatic cancer in 1969 demonstrated that this method can apply through bilateral injection of ethanol in a procedure called chemical splanchnicectomy or ethanol celiac plexus neurolysis (ECPN). CPB interrupts signal transfer rapidly, so that the result of CPN may remain for years [6, 11].

Despite the result of similar researches in the past two decades, neurolytic celiac plexus block (NCPB) significantly decreases chronic pancreatic cancer pain, by decreasing both reported pain scores and reducing postsurgical opioid intake [9].

Different methods of NCPB including the retrocrural, transaortic, and bilateral chemical splanchnicectomy which were performed intraoperatively or through thoracoscopy have been compared in a few studies, but they all showed considerably efficient outcome [5, 12, 13].

Although NCPB has been found as the preferred method due to its less invasiveness and more positive outcomes, few complications have been reported such as local pain (in 96%), diarrhea (in 44%) and hypotension (in 10%). Serious adverse effects such as neurologic (lower extremity weakness and paresthesia, epidural anesthesia, and lumbar puncture) and nonneurologic symptoms (pneumothorax, shoulder, chest and pleuritic pain, retroperitoneal bleeding, urinary retention, gastroparesis, bowel perforation, anterior spinal artery syndrome, aortic dissection or pseudoaneurysm, and hematuria) were rarely reported [5, 9, 13].

The current study is aimed to evaluate the efficacy of intraoperative chemical splanchnicectomy in cases with resectable upper abdominal masses who underwent surgical resection to provide immediate and long-life postoperative pain palliation for them. We decided to compare the result of celiac ganglion blockage during gastric and pancreatic mass resection and compare pain-relieving trend with the control group.

## 2. Materials and Method

This study aims to evaluate the effect of intraoperative chemical splanchnicectomy on pain intensity during first four months after surgical resection of gastric and pancreatic masses.

In order to compare the result of chemical splanchnicectomy between those with pancreatic masses with gastric masses and separately with those who did not receive intervention, 4 subgroups of patients were expected. Calculated sample size by Altman Nomogram and comparison of two means equation equals 21 cases for each subgroup and total number of 84 (Assuming alpha = 0.05, beta = 0.2, sigma = 3, and effect size = 3).

All patients with approved diagnosis of resectable pancreatic or gastric cancers who had referred to general surgery ward of Firoozgar Hospital during March 2015- September 2016 were involved.

Patients with unresectable pancreatic or gastric cancer, age less than 15 or more than 80, history of prolonged consumption of opioids, NSAIDS, analgesic and antidepressant drugs or opium addiction, cardiac or pulmonary diseases, sensitivity to opioid drugs, psychological disorders, abnormal arterial blood gas analysis, and those who underwent neoadjuvant chemotherapy were not included.

Patients who were dissatisfied with the procedures, expired before 4 months after operation or experienced surgical complications or any other signs that resulted in disability to complete pain scale questionnaire (such as loss of consciousness) or had prolonged postoperative hospitalization (more than 15 days) due to surgical complications were also excluded.

Throughout this study, all ethical considerations were respected. Previous studies have approved that chemical splanchnicectomy cannot cause serious harms to patients and has no concerning side effect. All patients agreed to involve in the study, and informed consent was obtained for probable intervention. The project has been submitted to the Iran randomized controlled trial system with submission code of IRCT2015022821269N1. Final participants with each of gastric or pancreatic masses were randomly divided into intervention and control subgroups.

The control group (including 24 cases of gastric and 19 cases of pancreatic cancers) did not receive any intervention.

For the intervention subgroups (24 cases of gastric and 23 of pancreatic cancers), the surgeon applied alcohol neurolysis.

While doing the procedure, surgeons had to gently find celiac ganglia on both sides of the celiac and superior mesenteric arteries which locate medial to the adrenal glands and anterior to the diaphragmatic crura. The surgeon retracted lesser curvature caudally, incised the avascular area of hepatogastric ligament, and put his left index finger on the splenic artery and the left third finger on the common hepatic artery while steadying their hand by abutting their thumb to the left lateral aspect of the aorta and straddled it and then palpated the celiac trunk to find the exact place and fixed it for injection (at the right position, the pulse of splenic artery and thrill of common hepatic artery are palpated by left index and third fingers of the left hand, respectively). Then, 20 ml of a 50% ethanol (German Merk Company; Art Number: 1.00983.2500) diluted in normal saline was injected to each of celiac ganglions at both sides of the suprapancreatic aorta by the right hand after aspiration of the syringe. The operation site was compressed by antibleeding packs.

During injection, all vital signs were under strict control, and in the presence of changes (if any), surgeons stopped injection and anesthesiologist resuscitate patient. Adrenaline and Atropine were used in case of anaphylactic shock and bradycardia, respectively. In order to palliate postsurgical pain, all patients received 25 mg pethidine 3 times per day, and extra amount was given when necessary. During the follow-up period, all patients were under direct control and received appropriate treatments if needed.

To eliminate operator bias, the study was designed as a double-blinded clinical trial so the surgeon who was not involved in the study injected solution to random cases. Neither the researcher nor the patient was aware of the injected solution. Researchers asked patients to evaluate their pain intensity at given times including one day before surgery, one, two, and four days after surgery, discharge time, one month, two months, and four months after surgery with the Visual Analogue Scale (VAS) for pain. To increase accuracy of their answers, the Wonge–Baker Face Pain Scale was also used.

At each interval, reported scores were then analyzed by SPSS version 22 by the repeated measure ANOVA test. To eliminate the bias effect, we subtracted before surgery pain scores from all follow-up times.

## 3. Results

**Figure 1:**
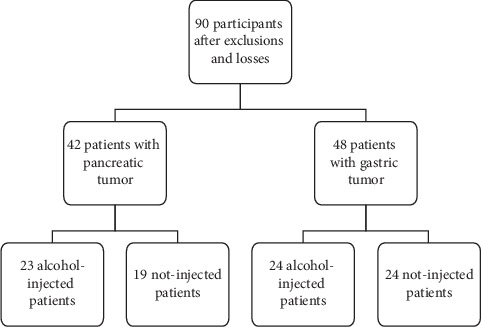
Allocation of participants in surgery subgroups.

From February 2015 to December 2015, 118 patients with resectable pancreatic or gastric tumors were recruited for randomization. Follow-up measurements were successful in 90 participants (28 were excluded: 12 lost to follow-up, 8 passed out, had long term hospitalization, and 2 underwent resurgery). The 90 were allocated in four surgery/intervention subgroups (Figure 1). Male to female proportion and mean age were not different among the surgery subgroups (*p* > 0.05) (Table 1).

**Figure 2:**
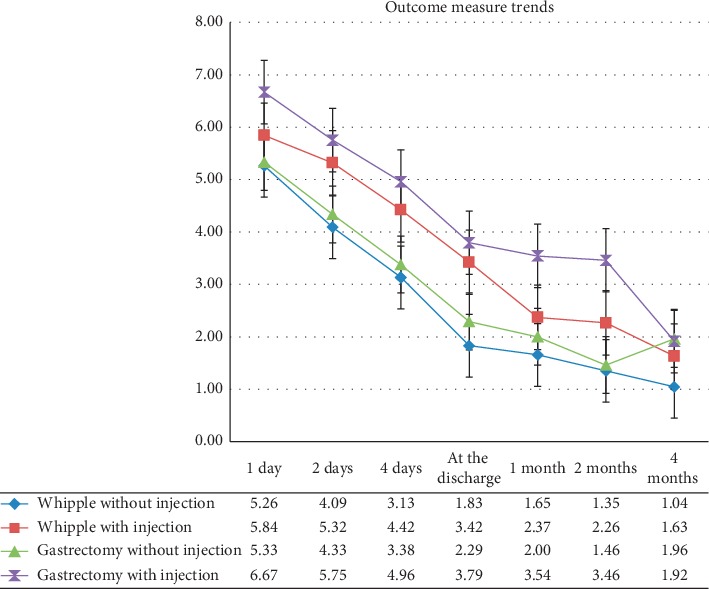
Trend of pain score during the 4 months after surgery.

Before the surgery, there were no differences between the pain of the injected and not-injected subjects in both Whipple (*p*=0.57) and Gastrectomy (*p*=0.12) groups. Mean pain scores in each follow-ups for the intervention subgroups are displayed in Table 2. Using the repeated measures ANOVA test, the overall postsurgery pain of injected Whipple and gastrectomy subgroups was lower than the noninjected Whipple and gastrectomy subgroups (*p* < 0.001). The pain modifying effect of the injection was not different between Whipple and gastrectomy groups (*p*=0.125) (Figure 2). There was no interaction between surgery (Whipple or gastrectomy) and intervention variables on pain score (*p*=0.875).

## 4. Discussion

Almost 70% of patients suffering from end-stage malignancies are complaining about intolerable moderate to very severe chronic discomfort. Most of patients with any advanced cancer experience episodes of visceral or bone pain [7], and 50 to 65 percent of patients with gastric cancer complain about abdominal pain at diagnosis [14].

In patients with pancreatic cancer, widening of pancreatic capsule due to mass lesion results in midepigastric pain, with radiation to the mid-back or lower-back region in up to 90 percent of patients.

According to the few times of life expectancy, palliation of symptoms is assumed as the primary goal of treatment for both operable and inoperable patients of these cancers [6].

Based on latest the World Health Organization guideline for pain management in pancreatic cancer palliation, use of analgesic drugs is the first-line treatment while recent studies revealed that complete pain relief cannot be achieved without invasive procedures. Nowadays, by addition of celiac plexus blocks and neurolysis, splanchnicectomy, and intrathecal therapies, overuse of drugs and their unwanted side effects decrease [7, 9].

There are two prominent reasons for choosing celiac ganglion to block: first, the role of thoracic splanchnic nerves in conducting pain sensation caused by the upper abdominal masses around the celiac ganglion; second, the splanchnic nerves are accessible and have a more predictable anatomical location and are not as closely associated with vital vascular and neurological structures [7, 15].

This study was designed to evaluating the effects of intraoperative chemical splanchnicectomy on relieving pain from gastric and pancreatic masses, and to achieve this goal, we applied this method in cases with operable masses to avoid the bias effect of pain control resulted by tumor resection itself.

Three sets of the sympathetic ganglia of celiac plexus constitute a relay station for visceral afferent nerve fibers are responsible for transferring pain signals from all pelvic and abdominal viscera. Outcomes and efficacy of celiac ganglion block in pain management are discussed in numerous studies [5, 16, 17]. Blockage of these ganglions was first applied by Kapsis in 1919 with very simple procedure.

The early experiences with image-guided celiac block were aided by angiography and fluoroscopy; later, the CT-guided chemical splanchnicectomy was emerged as the modality of choice for this procedure because it improves both precision in accuracy of injection and decreases risk of unintended faults [7].

Mallet-Guy performed the first left splanchnicectomy in 1942 through laparotomy in order to cure intractable pain due to chronic pancreatitis.

The first randomized controlled double-blinded trial estimating the benefits of intraoperative splanchnicectomy through laparotomy was performed by Lillemoe et al. in unresectable pancreatic masses. They significantly approve longer duration of pain relieving in the intervention group [6, 9].

Flanigan et al. also recommended open phenol splanchnicectomy at the time of initial laparotomy in patients with advanced intra-abdominal neoplasm [18].

A meta-analysis of 24 studies including 1145 patients conducted by Eisenberg E et al. showed 87% of pain relief in the short-term period (first 3 months). Most studies were able to provide long-term outcomes (≥3 months) from fewer patients [9, 13].

Raj et al. also applied celiac ganglion ablation patients with poor controlled chronic benign or malignancy-related abdominal pain and achieved short-term and long-term pain reduction in 85% of their cases [19].

Garcea et al. showed statistically significant benefits as well, including decrease in pain degree, opioid use, and hospital admissions for pain control, healthier mental status, and better perception of general well being [15].

Here, we applied intraoperative alcohol neurolysis with 50% ethanol injection to celiac ganglions with its safety and efficacy has approved in previous studies. By applying this easy and inexpensive intervention during the surgery, no further procedures are needed after massive abdominal surgeries.

Unfortunately, reported effects of alcohol splanchnicectomy are not permanent. Most of our patients had experienced moderate to severe pain recurring before death. Regarding that, these poor prognoses are susceptible to malnutrition, pneumonia, deep vein thrombosis, and other complication of immobility; relieving pain through much of their limited life expectancy is worth this invasive procedure [6].

It is expected that improving quality of life in patients with end-stage gastric or pancreatic cancers with controlling pain and a decreasing the demand on narcotic analgesics prolong their survival.

There are few self-limiting complications reported such as prolonged recovery period, mild and transient orthostatic hypotension, diarrhea, complications caused by trauma during the procedure, and local pain. The major uncommon but concerning complications include pneumothorax, chest pain, hematuria, hiccoughing, and neurological symptoms such as epidural anesthesia, weakness, and paresthesia of the lower limbs that need further workups and attention.

A case report in Japan suggested not applying alcohol neurolysis in cases with atherosclerosis of collaterals arteries or history of spinal cord infarction [20].

This method would not benefit those with alternative pathway carrying abdominal pain and anatomical variations, and predicted outcome does not achieve if the amount of alcohol injection or the placement of needle are not correct [7].

Besides all achievements in splanchnicectomy, there is still controversy surrounding this method. Some authors believe significant decrease in narcotic consumption is not reported following celiac block, and this probable outcome is not worth applying such invasion [21].

An Italian clinical trial study compared efficacy of celiac plexus blockage versus analgesics, decreasing of pain score was seen in both groups but there was no statistically significant difference between the two.

Through a double-blinded, randomized, controlled trial, Wong et al. reported long-term pain relief after celiac ganglion block but could not significantly demonstrate its effect on quality of life, opioid drug use, and survival [16].

Even though in one published study, bilateral chemical splanchnicectomy was not recommended since it seemed to have no effect or its effect may have been masked by the surgery [11].

A review of articles persist that neurolytic block should apply only when opioids fail to control the pain or side effects of opioids consumption leads to more organ damage [7].

This study was undertaken to confirm the efficacy of intraoperative chemical splanchnicectomy and suggest this method as an efficient, safe, and inexpensive way to palliate pain after upper abdominal major surgeries. The efficacy was approved for Whipple surgery. Regarding that celiac ganglions are also known as the origin of pain caused by gastric masses, we aimed to compare the result for total gastrectomy and Whipple.

The result of this study revealed the significant effect of pain palliation in both groups in comparison with the control group of each, but the difference was a bit more at Whipple surgery. During first four months, the difference disappeared slightly and reported pain degree in both the control and intervention group seemed the same. There are two main descriptions for this evidence; first is local recurrence of tumor and the second is unfortunate nonpermanent effect of chemical splanchnicectomy. Since higher pain intensity is expected for local recurrence than observed, the first reason seems less accurate. Assuredly, tumor resection itself can cure the pain after few months (in order to eliminate the bias caused by tumor resection, we only included operable patients). However, since the life expectancy for end-stage patients is not much more than few months, we urge surgeons to provide them with safe, dignified, and reliable palliative care.

For further researches, it is suggested to follow long-time outcomes and its possible complications for other gastrointestinal massive surgeries. For more reliable outcomes, it is recommended to exclude cases whose pain relates to previous surgery or endoscopic procedures.

## 5. Conclusion

Intraoperative bilateral celiac ganglion ablation by injection of 20 cc 50% ethanol to each ganglion is an easy, effective, warranted safe, and inexpensive procedure recommended for all operable gastric or pancreatic masses to palliate the pain degree. Although this method has short-time effect, it can be helpful for patients suffering from postsurgical pain and more preferred than undergoing percutaneous or endoscopic chemical splanchnicectomy after surgeries.

## Figures and Tables

**Table 1:** Distribution of gender and age among subgroups.

	Numbers	Females	Age
	*N* (%)	*N* (%)	Mean years (SD^*∗*^)
Whipple surgery			
Injected	23 (25.6)	9 (33)	56 (11)
Not-injected	19 (21.1)	6 (22)	52 (14)
Gastrectomy			
Injected	24 (26.7)	5 (19)	60 (14)
Not-injected	24 (26.7)	7 (26)	55 (11)
Total	90 (100)	27 (100)	56 (13)

^*∗*^Standard deviation.

**Table 2:** Pain score trends.

	Before surgery	One day after surgery	Two days after surgery	Four days after surgery	At discharge	One month after surgery	Two months after surgery	Four months after surgery
Whipple								
Injected	3.17 (0.47)^*∗*^	5.26 (0.34)	4.09 (0.61)	3.13 (0.47)	1.83 (0.37)	1.65 (0.32)	1.35 (0.24)	1.04 (0.24)
Not-injected	2.89 (0.56)	5.84 (0.63)	5.32 (0.61)	4.42 (0.56)	3.42 (0.52)	2.37 (0.58)	2.26 (0.34)	1.63 (0.47)
Gastrectomy								
Injected	3.58 (0.48)	5.33 (0.52)	4.33 (0.52)	3.38 (0.48)	2.29 (0.47)	2.00 (0.32)	1.46 (0.32)	1.96 (0.31)
Not-injected	2.50 (0.46)	6.67 (0.41)	5.75 (0.48)	4.96 (0.46)	3.79 (0.40)	3.54 (0.47)	3.46 (0.43)	1.92 (0.43)

^*∗*^ *Note.* Values are reported as mean (standard deviation).

## Data Availability

The data used to support the findings of this study are available from the corresponding author upon request.

## References

[1] Tomasello G., Ghidini M., Liguigli W., Ratti M., Toppo L., Passalacqua R. (2016). Targeted therapies in gastric cancer treatment: where we are and where we are going. *Investigational New Drugs*.

[2] Pasechnikov V., Fedorov E., Kikuste I. (2014). Gastric cancer: prevention, screening and early diagnosis. *World Journal of Gastroenterology*.

[3] Colquhoun A., Arnold M., Ferlay J., Goodman K. J., Forman D., Soerjomataram I. (2015). Global patterns of cardia and non-cardia gastric cancer incidence in 2012. *Gut*.

[4] Qiubo Zhang L. Z., Chen Y., Lian G. (2016). Pancreatic cancer epidemiology, detection, and management. *Gastroenterology Research and Practice*.

[5] Stefaniak T., Basinski A., Vingerhoets A. (2005). A comparison of two invasive techniques in the management of intractable pain due to inoperable pancreatic cancer: neurolytic celiac plexus block and videothoracoscopic splanchnicectomy. *European Journal of Surgical Oncology (EJSO)*.

[6] Keith D., Lillemoe M. D., John L. (1993). Chemical splanchnicectomy in patients with unresectable pancreatic cancer: a prospective randomized trial. *Annals of Surgery*.

[7] Tam A., Ahrar K. (2007). Palliative interventions for pain in cancer patients. *Seminars in Interventional Radiology*.

[8] Lică I., Jinescu G., Pavelescu C., Beuran M. (2014). Thoracoscopic left splanchnicectomy—role in pain control in unresectable pancreatic cancer. Initial experience. *Chirurgia (Bucur)*.

[9] Hameed M., Hameed H., Erdek M. (2010). Pain management in pancreatic cancer. *Cancers*.

[10] Malec-Milewska M. B., Tarnowski W., Ciesielski A. E., Michalik E., Guc M. R., Jastrzebski J. A. (2013). Prospective evaluation of pain control and quality of life in patients with chronic pancreatitis following bilateral thoracoscopic splanchnicectomy. *Surgical Endoscopy*.

[11] Lavu H., Lengel H. B., Sell N. M. (2015). A prospective, randomized, double-blind, placebo controlled trial on the efficacy of ethanol celiac plexus neurolysis in patients with operable pancreatic and periampullary adenocarcinoma. *Journal of the American College of Surgeons*.

[12] Ischia S., Ischia A., Polati E., Finco G. (1992). Three posterior percutaneous celiac plexus block techniques a prospective, randomized study in 61 patients with pancreatic cancer pain. *Anesthesiology*.

[13] Eisenberg E., Carr D. B., Chalmers T. C. (1995). Neurolytic celiac plexus block for treatment of cancer pain. *Anesthesia & Analgesia*.

[14] Mansfield Y. J., Crane C. H. (2003). *Clinical Manifestations*.

[15] Garcea G., Thomasset S., Berry D. P., Tordoff S. (2005). Percutaneous splanchnic nerve radiofrequency ablation for chronic abdominal pain. *ANZ Journal of Surgery*.

[16] Wong S. D., Carns P. E., Wilson J. L. (2004). Effect of neurolytic celiac plexus block on pain relief, quality of life, and survival in patients with unresectable pancreatic cancer: a randomized controlled trial. *JAMA*.

[17] Kawamata M., Ishitani K., Ishikawa K. (1996). Comparison between celiac plexus block and morphine treatment on quality of life in patients with pancreatic cancer pain. *Pain*.

[18] Flanigan D. P. (1978). Continuing experience with palliative chemical splanchnicectomy. *Archives of Surgery*.

[19] Raj P. P., Sahinler B., Sahinler B. (2002). Radiofrequency lesioning of splanchnic nerves. *Pain Practice*.

[20] Maher J. W., Johlin F. C., Pearson D. (1996). Thoracoscopic splanchnicectomy for chronic pancreatitis pain. *Surgery*.

[21] Akhan O., Ozmen M. N., Basgun N. (2004). Long-term results of celiac ganglia block:correlation of grade of tumoral invasion and pain relief. *American Journal of Roentgenology*.

