# Corrigendum to “Pain Relieving Effect of Intraoperative Chemical Splanchnicectomy of Celiac Ganglions in Patients with Resectable Pancreatic or Gastric Masses: A Randomized Clinical Trial”

**DOI:** 10.1155/2021/9536729

**Published:** 2021-02-22

**Authors:** Jalal Vahedian, Amir Saraee, Massoud Baghai Wadji, Paniz Motaghi, Kia Vosoughi, Saeed Safari, Abdolhamid Chavoshi Khamneh

**Affiliations:** ^1^Iran University of Medical Sciences, Firoozgar Hospital, Tehran, Iran; ^2^Tehran University of Medical Sciences, Shariati Hospital, Tehran, Iran

In the article titled “Pain Relieving Effect of Intraoperative Chemical Splanchnicectomy of Celiac Ganglions in Patients with Resectable Pancreatic or Gastric Masses: A Randomized Clinical Trial” [1], two authors were mistakenly omitted from the author list. The corrected author list and affiliations are given above.

## References

[1] Vahedian J., Saraee A., Baghai Wadji M., Safari S., Chavoshi Khamneh A. (2020). Pain Relieving Effect of Intraoperative Chemical Splanchnicectomy of Celiac Ganglions in Patients with Resectable Pancreatic or Gastric Masses: A Randomized Clinical Trial. *Pain Research and Management*.

